# Modeling Release Scaffolds for Spinal Cord Tissue Regeneration After Injury Using COMSOL Simulation

**DOI:** 10.3390/nano16100638

**Published:** 2026-05-21

**Authors:** Tasnim Hasan Al Dabbas, Ayat Bozeya, Ali Al Dabbas

**Affiliations:** 1 Institute of Nanotechnology, Jordan University of Science and Technology, Irbid 22110, Jordan; dabbass14@gmail.com; 2 Jordan Atomic Energy Commission, Shafa Badran, P.O. Box 70, Amman 11934, Jordan; ali.dabbas@jaec.gov.jo

**Keywords:** spinal cord, scaffold, reduced graphene oxide, COMSOL, Michaelis–Menten kinetics, glutamate

## Abstract

The current study illustrates the modeling of a biocompatible poly γ-glutamic acid (PGA)–chitosan–rGO nanocomposite hydrogel scaffold, which showed a promising novel scaffold for stimulating central nerve regeneration that addresses the shortcomings of recent therapies and improves tissue engineering, controls inflammation, and restores lost functions after spinal cord injuries (SCIs). In the implementation part of the study, the COMSOL program’s top-notch modeling of a detailed investigation of how a scaffold’s in vivo diffusion affects injured neurons. Michaelis–Menten kinetics is used to characterize the enzyme process of releasing the outer covering shell of the scaffold, PGA, from a biomaterial matrix to the nerve cell. Results suggested that the injectable hydrogel scaffold theoretically reduces extracellular glutamate concentrations, presenting a potential mechanism to mitigate localized excitotoxicity. Future in vivo experimental validation is required to determine if this reduction prevents neural cell death

## 1. Introduction

Worldwide, spinal cord injuries (SCIs) are among the leading causes of mortality and permanent disability. At present, there are no tried-and-true neuroprotective treatments for SCIs. This may be traced back, at least in part, to the intricate damage processes. Mechanical trauma generates immediate tissue damage (primary injury), whereas metabolic alterations lead to more gradual cell death (secondary injury) [1]. Despite substantial heterogeneity in pathophysiology, numerous conditions have common damage mechanisms in a number of essential regions. Some cells are killed off by the mechanical trauma of SCIs, but many more are killed off by the metabolic alterations brought on by the damage itself (secondary injury) [2]. In the case of reestablishing axonal connections after damage or sickness, the neuroscience dogma states that nervous system components have a low regenerative ability. The decades of study in tissue engineering have brought forward fresh viewpoints demonstrating viable alternatives to the previously divisive debate. Successfully regenerating brain systems is simply a question of time, as shown by the use of the tissue engineering paradigm, which includes cell transplantation and the creation of biomaterials to replace native extracellular matrix (ECM) [3].

### 1.1. After Spinal Cord Injury

Mechanical trauma causes cellular strain and plasma membrane damage, which triggers a cascade of secondary effects [1], including localized acidosis, hyperpolarization of nearby water molecules, and the release of excitatory amino acids. Each of these primary modifications sets off a cascade of secondary ones that ultimately causes the surrounding healthy cells to become damaged. Macrophages and other immune cells may enter the brain and spinal cord after being first damaged during physical assault [2]. There may be a second peak of inflammation up to 2 months post-injury, according to recent research [3,4], and long-lasting neuroinflammatory alterations have also been observed. After SCI, there is enough glutamate released to start causing white matter damage. Immune cells migrate across the broken blood–spinal cord barrier (BSCB) to clean up the dead tissue. Because of this, more neurons and oligodendrocytes die, causing secondary damage. Through the formation of a rigid scar matrix, astrocytes rebuild a physical barrier, which serves to protect the wounded tissue from future injury. The combination of these factors reduces the injured site’s ability for regeneration. Since oligodendrocyte densities along the fiber track were not substantially altered after 72 h, most glutamate-induced oligodendrocyte death occurred within the initial 24 h acute window, before secondary neuroinflammatory cascades are fully manifested [5].

Spinal cord injury releases glutamate above toxicity in cultured neurons but below in vivo. Glutamate neurotoxicity thresholds and extracellular glutamate concentrations vary after spinal cord injury. In physiologic settings, the concentration ratio extrapolated to zero flow to the concentration in samples taken at 2 mL/min demonstrates that the reuptake mechanism is well-suited to maintain glutamate levels in the normal 1–5 μM range. This high-affinity method cannot quickly reduce glutamate in high synaptic clefts > (500) μM [6]. Liu et al. [7] found 0.53 mM glutamate after spinal cord impact damage. These values are greater than the cultured neuron toxicity threshold (0.05–0.10 mM) but lower than the in vivo glutamate neurotoxicity threshold. Excessive excitatory glutamate exposure may cause cell death until it reaches the in vivo glutamate neurotoxicity threshold [6], which means the neural cells were lost early but were not recognized, and the astrocyte had yet to be invoked. The effects of glutamate on T cells are concentration-dependent, although even low levels may have an impact; T cells are a kind of white blood cell that helps defend the body against infection. Glutamate by itself, at low physiological concentrations of 10^−8^ M to 10^−5^ M and via its several types of glutamate receptors (GluRs), activates many key T-cell functions in normal human T cells, which affect other cells, and dendritic cells release glutamate, which strongly influences T cells. These findings imply that glutamate strongly affects normal and autoimmune pathogenic T cells [8].

### 1.2. Enzyme Michaelis–Menten Kinetics

Understanding how astrocytes maintain glutamate homeostasis and govern central nervous system (CNS) glutamate uptake and release might help explain CNS disorders involving glutamate excitotoxicity [9]. Extracellular glutamate increases glutamine synthetase (GS) activity and astrocyte glutamine release [10]. Astrocytes protect neurons by removing excess glutamate and producing glutamine for neurons via the metabolic pathway, mainly by GS [11].

Despite a vast improvement in our knowledge of neuropathophysiology, we still lack effective methods for hastening axon development and restoring full function during neurorepair. Neurons are the most important portion of both neurological [12,13] systems, yet they are unable to undergo mitosis, and the glial cells that support them can only divide so many times. After a nerve is cut, not only does it take a long time to mend, but the distal ends also deteriorate because of protease activity. As a result, the human nervous system cannot recover from injury or sickness on its own and is very susceptible to such harm [14].

### 1.3. Is It Possible to Rapidly Remove Glutamate from the Injured Region of the Spinal Cord?

After spinal cord loss, the lamprey’s glutamatergic system heals. Fernández-López et al. showed that regenerated vertebrates, like the sea lamprey’s glutamatergic system, spontaneously restructure and renew their spines [15,16]. At 10 weeks post-injury (wpi), glutamatergic neurons/processes recovered nearly completely. Glutamate release after a lesion may kill neurons and oligodendrocytes [5,7,17], worsening the lesion’s prognosis. Short-term glutamate changes following SCI have been frequently reported [17,18], particularly in lampreys. Blanca et al. found that lamprey neurons produce a large amount of glutamate after SCI. One week after cord transection, astrocytes were labeled with glutamate, indicating functional glutamate absorption [18]. Long-term glutamatergic system changes are unclear. Fernández-López et al. [16] showed that lampreys lost all glutamate immunoreactivity immediately after a complete rostral and caudal cord transection to the injury site, suggesting excessive glutamate release. And after just 10–12 weeks, they may recover from total paralysis and return to swimming [19].

Proteins and polysaccharides make up the extracellular matrix (ECM), which is found between neuronal and glial cells. Due to its structural features, the ECM is a natural barrier that limits cell migration and the release of soluble particles. Modifying the crosslinking density of the gel matrix and the hydrogel porosity (mesh size) may regulate drug transport and release across the polymer network [20].

Hydrogels’ bioadhesion makes them useful as scaffolds for tissue regeneration or surgical adhesives because they attract and hold cells and tissues. Linker molecules generate chemical interactions between the gel matrix and its surroundings, either non-covalently or covalently [21,22], hence increasing cell/tissue adhesion.

Filling microscopic flaws like the cavities/cysts that occur after SCI or the gap between transected portions of the spinal cord is possible thanks to the injection of a liquid that, in vivo, solidifies into a physical or chemical gel. Before injection, injectable materials are fluid, but thereafter they solidify. Due to their injectable nature, hydrogels may be implanted in the body with minimal surgical incisions and entrap therapeutic molecules or cells at regions through syringe injection [23]. The gel might fill in the gaps and act as a regenerative template, attracting and concentrating healing cells and extracellular matrix at the site of damage [24]. Because the gel may be injected directly into the affected area, it is unnecessary to remove healthy tissue around the injury before treating it. The release of these agents may be regulated in space and time by a variety of methods, including incorporation into secondary release vehicles, such as micro- and nanoparticles [25,26] and covalent anchoring to the gel, followed by enzymatic release and so on [27].

Spinal cord injury patients may benefit from axonal regeneration and the suppression of scar formation after cellular interaction with injectable scaffolds. The direct attachment of in situ-forming scaffolds to the host enables both the restoration of spinal cord continuity and the infiltration of cells. The effects of several r(GO/alginate)-based MSC delivery techniques on a heart disease model were studied. Scientists sought to employ reduced graphene microgels in inflammatory bowel disease and other types of tissue degeneration in which oxidative stress plays a significant role [28,29,30,31].

In a few instances from the literature, to ensure the steady release of doxorubicin to tumor cells, a nanoparticle (NP) delivery system was developed. γ-poly-glutamic acid (PGA), graphene oxide (GO), and chitosan oligosaccharide (CO) make up the nanoparticle system. The GO-CO-PGA system was created by loading γ-PGA onto a composite made of graphene oxide and chitosan oligosaccharide in a covalent bonding reaction. The doxorubicin on the nanocarrier was claimed to be released slowly and steadily. With γ-PGA present, GO-CO-PGA-DOX was more effective at inhibiting proliferation in an in vitro cytotoxicity experiment with 3-[4,5-dimethylthiazol-2-yl]-2,5 (MTT) than when γ-PGA was missing. Most studies have concluded that γ-PGA might be widely used in medication administration in the near future [32].

Scientists investigated how peptides could revive damaged nerve cells. Enzymatic degradation protects neurons against glutamate excitotoxicity [33]. Glutamine synthetase enzyme (GS) converts glutamate from glutamatergic neurons to glutamine in astrocytes. Neurons take glutamine from astrocytes, which glutaminase converts to glutamate [11]. Understanding how astrocytes maintain glutamate homeostasis and govern CNS glutamate uptake and release might help explain CNS disorders involving glutamate excitotoxicity [9]. Extracellular glutamate increases GS activity and astrocyte glutamine release [10]. The amino acid GABA (gamma-aminobutyric acid) is produced from glutamate by the enzyme glutamate decarboxylase (GAD) in conjunction with pyridoxal phosphate (the active form of vitamin B6). Glutamate, the main excitatory neurotransmitter, is converted into GABA, the main inhibitory neurotransmitter, by this mechanism [34,35]. GABAergic neurons, so-named because of the glutamate-blocking amino acid (GABA) they generate, mostly reduce receptor activity in adult vertebrates. The medium spiny cell is representative of the inhibitory GABAergic cells found throughout the central nervous system [34]. When one of glutamate, adenosine triphosphate ATP, or ammonia is in short supply while the others are plentiful, glutamine synthetase exhibits Michaelis–Menten kinetics. Astrocytes protect neurons by removing excess glutamate and producing glutamine for neurons via the metabolic pathway, mainly by GS [11].

Glutamate transporters in the spinal cord remove glutamate from the synaptic cleft to preserve proper sensory transmission. Inhibition of glutamate transporters in the spinal cord activates inhibitory presynaptic group III metabotropic glutamate receptors, which reduce inflammatory pain, as shown in this research. Previous research has established a novel morphologic foundation for the functional involvement of spinal glutamate transporters in both normal sensory transmission and pathologic pain (including inflammatory pain) [36].

### 1.4. rGO as Novel Material for Neural Tissue Engineering

Scientists have made a significant discovery in the nanotechnology “gold rush”, thanks to graphene. Graphene nanomaterials have been used in neural tissue engineering applications due to their high electrical conductivity [37,38,39,40,41]. While previously, nanotechnology was primarily used in medical applications such as neurodegeneration, inflammatory neurological diseases, and neural tissue engineering, new uses have recently been discovered. Graphene-based nanomaterials have recently captured researchers’ interest due to their diverse applications [40]. Numerous biomedical applications are using graphene and reduced graphene oxide (rGO) because they have excellent physical properties, conductivity, and the ability to interact with neurons and neuronal circuits [37]. Nonetheless, rGO materials in humans must be administered prior to therapeutic delivery. Standardized parameters are necessary to assess the toxicity of rGOs in clinical trials, such as diagnosis and treatment. rGO biomedical applications deal primarily with solubility, biocompatibility, and selectivity. An intelligent understanding of cellular- and atomic-level issues is critical. With regard to biomedical applications, a natural rGO change can help by helping to overcome the challenges of therapeutic delivery [40,42].

The University of Manchester’s Department of Physics has been growing one-atom-thick two-dimensional crystal graphene since 2004.

rGO microfibers have unique properties such as cost-effectiveness, mechanical strength, flexibility, nontoxicity, lightness, and excellent cytocompatibility, as well as the ability to control neural stem cell (NSC) differentiation into neurons and support NSC growth, allowing for the fabrication of high-quality carbon materials with specific carbon-to-oxygen (C/O) ratios. Furthermore, rGO compounds are more water-soluble than GO compounds, resulting in better squandering. As a result, nanostructured rGO microfibers provide a more vigorous substrate for NSCs than 2D graphene films and tissue culture plates [37,38,43,44,45].

The toxicity of GO has been shown to be less toxic than graphene, and less toxic than rGO and hydrogen-G [44]. As noted by Zhang et al. [28], studies have shown no significant neurotoxicity signals, including no clinical convulsive signs or engine abnormalities, when treating rats with rGO flakes. However, a short-term decrease in coordination between neuromuscular connections has been observed in mice with rGO nanosheet administered orally [46,47].

Zhu’s studies showed no direct results at low levels of significant cytotoxicity. In contrast, in studies pertaining to size, big flakes have been found to be more toxic than smaller nanosheets. The aggregate effect density was also studied, and it was shown that those aggregated were more toxic than G solutions that were highly dispersible [45,46,48]. A Live/Dead Toxic Cell Test of rGO microfiber, conducted by Guo et al., in which rGO microfiber cytotoxicity was tested under multiple conditions of tissue cultivation plates and 2D graphene films as controlling test, was performed following three days of culturing neural stem cells. The results show that ~95.8% of the NSCs live on nanostructured rGO microfiber after culture, while ~94.1% was observed on the tissue culture plate and ~93.5% on 2D graphic film; for the NSCs cultured with nanostructured rGO microfiber, there was no apparent cytotoxicity [38,45,49].

Due to their remarkable properties, such as flexibility and versatility in fabrication, variety in composition, high tunability in physical, chemical, and biological properties, high moldability in shape, and excellent biocompatibility and similarity to native (ECM), hydrogels are promising materials of great importance in biomedical fields and have been extensively studied in academic and industrial research [20,21,50,51,52,53].

Despite the fact that graphene oxide is composed of the same atoms as human organs, tissues, and cells, graphene oxide’s bi-dimensionality interacts with blood proteins and biological membranes, causing thrombogenicity and immune cell activation [54]. Strong π-π interactions make macroscopic constructions built from dispersible graphene sheets bio-persistent, despite the sheets’ biodegradability [55]. rGO nanoparticles were observed to be somewhat toxic [18]. Moreover, the tissue-material contact is unstable; the graphene films distort and are depressed after insertion. These depressions accumulate blood, bodily fluids, and proteins, triggering host cell aggregation, including macrophages. These macrophages eventually cause myofibroblast populations to develop, with myofibroblasts exerting contractile force [55]. Graphene-based nanocomposites have been shown in a growing number of recent studies to be effective platforms for promoting neural stem cell adhesion, proliferation, differentiation, and neural regeneration. This evidence strongly shows that graphene nanocomposites are useful in the field of regenerative medicine for the nervous system [14].

### 1.5. The Study Hypothesis

This study involves simulating an rGO-based nanocomposite scaffold using COMSOL. The scaffold is developed through a simple electrostatic layer-by-layer self-assembly nanosphere approach, combining poly-glutamic acid and chitosan polymers into a hybrid hydrogel model. The outer shell of the scaffold consists of poly-glutamic acid, which demonstrates remarkable efficiency in promoting tissue regeneration after neuronal cell damage and exhibits good biocompatibility.

The main objective of this study is to investigate a more effective use of biomaterials in promoting tissue regeneration. By integrating molecular biology and engineering strategies, scaffold delivery systems are incorporated into materials to enhance tissue regeneration.

Since astrocytes exhibit significant glutamate absorption after SCI, like what is seen in lampreys after cord transection for only one day, the present study aims to examine the effect of outer surface glutamate at a concentration that is greater than the in vivo glutamate neurotoxicity threshold of >0.53 mM. As such, glutamate will be intensively injected in the injured spinal cord within the first 72 h after edema to increase astrocyte uptake as quickly as possible, in order to safeguard neurons by removing glutamate and making sure that the prepared scaffold penetrates the damaged neurons before the immune system is stimulated without toxic interferences. To create an identical environment to that of a lamprey’s in vivo conditions following a spinal cord injury, it is assumed that ammonia (NH_3_) concentration is constant and is only considered in conversion rates. Glutamate and glutamine are nearly identical molecules, so we will assume that glutamate and glutamine are nearly identical. Glutamate substrates will have the lowest concentration at a limited level, allowing glutamine synthetase to exhibit Michaelis–Menten kinetics. The detailed reaction and transport description used in this model allows for the investigation of many design parameters relevant to bioengineering.

## 2. Computational/Simulation Framework

### 2.1. COMSOL Multiphysics

The objective of this section is to provide a foundational understanding of COMSOL Multiphysics by explaining how to set up fully determined models within the software. Being able to witness COMSOL Multiphysics in motion is far more illuminating than reading about it or looking at screenshots of it in action. This section provides context for the specific COMSOL Multiphysics features that the reader should evaluate based on their own modeling goals. These features include the simulation of in vivo drug release, as well as a wide range of diffusion applications across multiple domains. COMSOL software did an excellent job of handling passive diffusion, which takes into consideration the density differential of neurons with distinct functions to explain how therapeutic substances move in vivo, and how different species of these substances diffuse at different rates through nerve cells and other porous cells.

### 2.2. Modeling and Fundamentals

**Figure 1:**
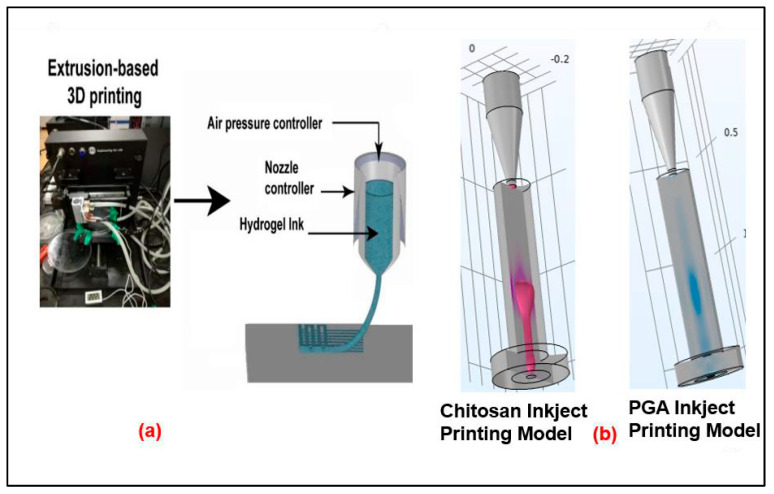
Modeling software preparation for Inkject printing: (**a**) The extrusion-based 3D printing method [57], (**b**) modeling software preparation of Injection-able using the COMSOL Multiphysics. Left—chitosan, the first layer; right—PGA, the outer layer in the module.

These simulation studies were conducted in COMSOL Multiphysics software version 6.0. The extrusion-based 3D printing method (Figure 1) works by depositing ink to build the 3D structure layer by layer. The fluid dynamic modeling of the extrusion process serves strictly to verify the structural viability of the core–shell formation prior to injection. Confirming this stable layer-by-layer assembly justifies the initial geometric boundary conditions applied in the subsequent in vivo biological release model.

**Figure 2:**
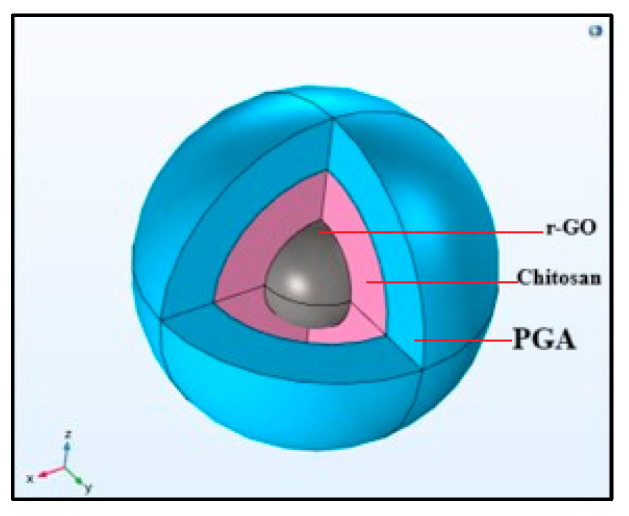
Two-layer-by-layer presentation of the research model, with the gray core representing an rGO composite covered by two layers. The first inner layer in pink is chitosan, while the outside layer in blue is PGA.

However, the flow characteristics and design improvement of polyelectrolytes, PGA, and chitosan-based inks assembled layer-by-layer around the rGO composite were studied with the use of a numerical simulation based on 3D printing with chitosan and PGA (Figure 2).

As a result, researchers looked at the mechanism and scaling characteristics of PGA and chitosan-based ink at a range of speeds and viscosities. Hydrogel 3D printing often involves other factors (such as substrate temperature), which is critical because the thermal gradient directly dictates the sol–gel transition kinetics, ensuring the extruded filament maintains its structural fidelity upon deposition. However, with a new material like chitosan hydrogel, the substrate temperature was maintained at a steady 60 °C. This specific temperature was selected to force the immediate thermal gelation of the chitosan solution upon contact, preventing structural deformation during the layer-by-layer assembly. For this reason, the chitosan ink for this innovative chitosan hydrogel has to be a stable solution held at a low temperature (5 °C) throughout the 3D printing process. Chitosan, on the other hand, will self-assemble into a gel when heated. Using this idea as a starting point, we analyzed how changing the concentration and velocity parameters in the CFD simulation affected the flow [56].

**Figure 3:**
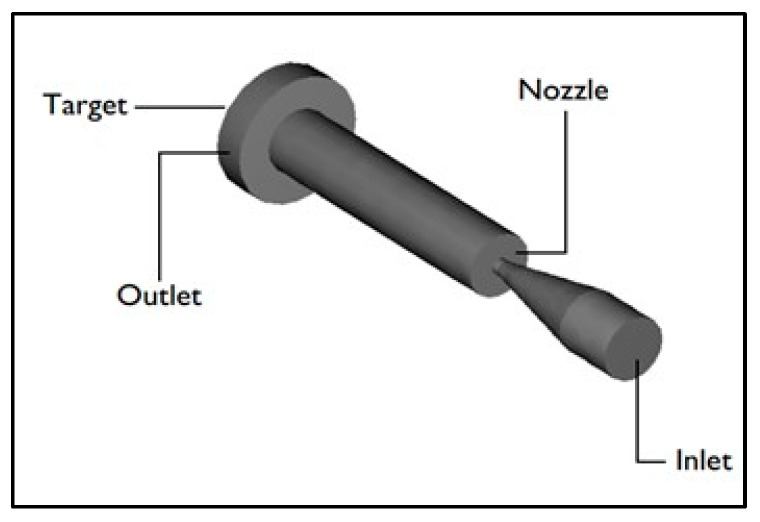
Image of 3D geometry of the Inkject.

For the chitosan-based ink and the PGA-based ink, the Weber number did not need verification since there was no fluid supply at the nozzle’s input (Figure 3). These are incompressible fluids, because the bond number B0 for them in this study is on the order of 1.0. The two experiments used the same COMSOL software to examine the effects of various kinds of ink and surface tension (but not gravity) on droplet mobility.

**Figure 4:**
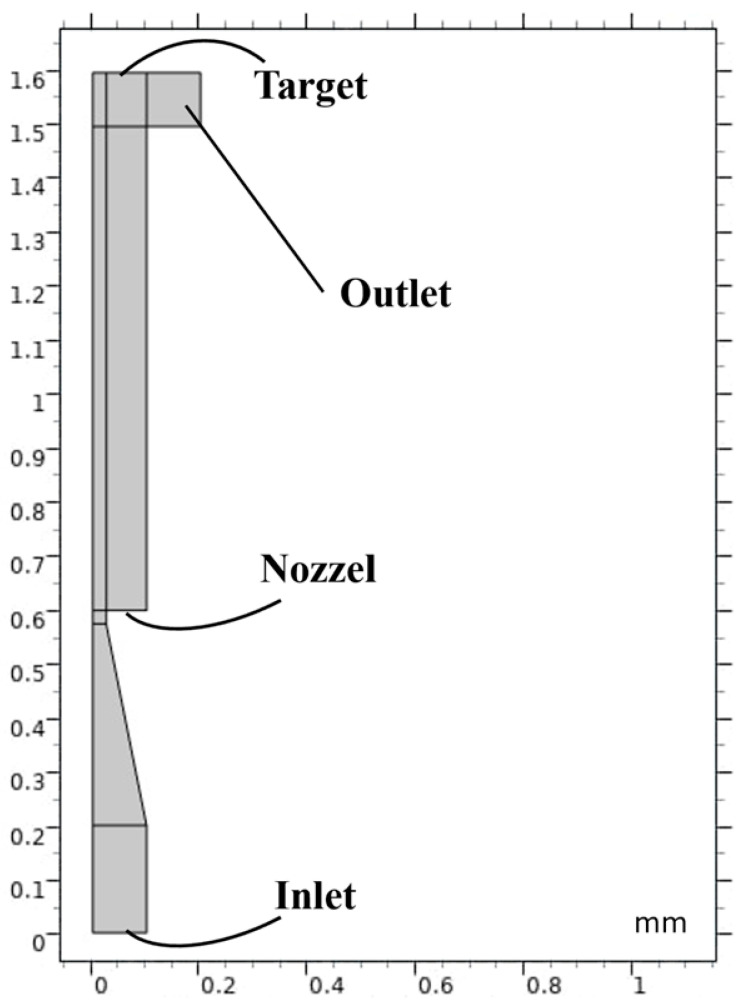
Geometry of the nozzle, two-dimensional model with Axis Symmetry.

To use a two-dimensional model with Axis Symmetry (Figure 4), ink is first loaded into the region between the ink supply and the nozzle. Ink is forced out of the nozzle by injecting more ink via the intake over a period of 2 × 10^−3^ s. A single ink droplet is released when the injection process ends, and it travels until it reaches its destination.

This study looked at how cell viability affects 3D bio-printing using a simulation model, examining the differences between screw- and syringe-based extrusion 3D printing methods. Finite element and adaptive mesh control methods are both used in this study. The simulation presented the results of numerical experiments applicable to issues in materials science. Both surface tension and mass conservation played significant roles in these situations. Two- and three-dimensional problems on both uniform and non-uniform grids are investigated. Convergence and conservation are investigated because of these numerical experiments, and the convergence and conservation properties are found to be satisfactory [57]. The fluid dynamic modeling of the extrusion process serves strictly to verify the structural viability of the core–shell formation prior to injection. Confirming this stable layer-by-layer assembly justifies the initial geometric boundary conditions applied in the subsequent in vivo biological-release model.

### 2.3. Boundary Conditions and Stability

In this study, the geometric domain was reduced to a 2D axisymmetric plane to optimize computational efficiency. The axial symmetric boundary conditions were mathematically formulated as zero flux (∂C/∂r = 0) at the symmetry axis (r = 0). To ensure numerical stability and grid independence, a mesh convergence study was conducted. The mesh was refined until the variation in the maximum concentration of glutamate was less than 1% between successive refinements. Similarly, a time-step independence test confirmed that a time step of 0.01 s was sufficient to capture the transient kinetics without numerical oscillation.

### 2.4. Microfluidic Hydrogel Delivery Mathematical Modeling

**Figure 5:**
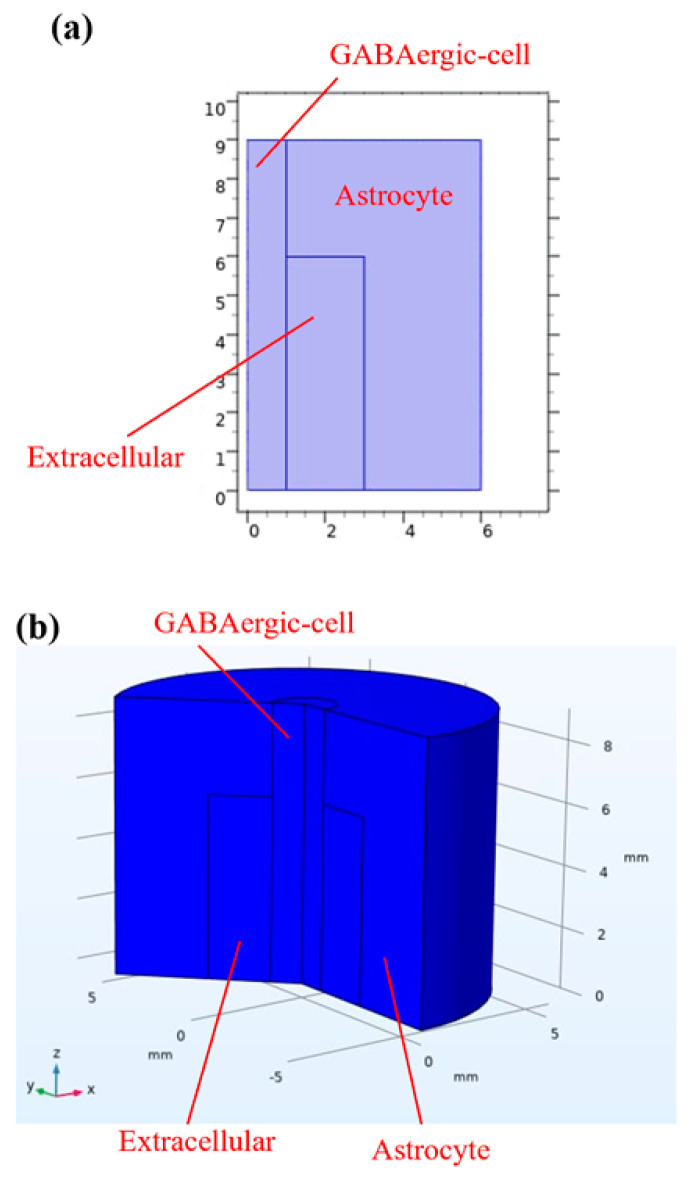
The equivalent modeling domains, with the regions being GABAergic cell tissue, astrocyte, and extracellular medium: (**a**) 2D by axial symmetry, and (**b**) the full 3D geometry.

The model (Figure 5) is structured around three key domains: astrocyte, extracellular cell, and nerve (GABAergic) cell. Its purpose is to facilitate the observation of glutamate and glutamine kinetics between a neuron and an astrocyte in a more straightforward manner. The study incorporates an rGO composite/chitosan polymer/poly (γ-glutamic acid) scaffold, which is released to the damaged cell tissue. Specifically, a nerve guide delivers a regenerating scaffold to the damaged area. The model stimulates detailed scaffold release kinetics, utilizing a rate expression that governs the dissociation/association process through enzyme catalysis.

The enzyme reaction is characterized by Michaelis–Menten kinetics [58,59,60,61]. It is characterized as closed with one loss and one input term, which compensate for each other. Glutamate (GLU) and glutamine (GLN) exchange in the neural cell. By the function of time, the glutamate lost in the extracellular space is immediately replaced by glutamine uptake from the blood in the astrocyte. Glutamate and glutamine are nearly identical molecules. Glutamate is taken up by astrocytes and converted to glutamine by the glutamine synthetase enzyme (GS). Astrocytes emit glutamine, which is reabsorbed by neurons and digested by glutaminase to glutamate. The conversion of glutamate to glutamine stops at about 90% of completion. The model assumes Ammonia (NH_3_) concentration is constant and only considered in conversion rates. Astrocytes require more than one adenosine triphosphate (ATP) molecule for each molecule of glutamate taken up. Finally, glutamate is dissociated in GABAergic cells to release the encapsulated r(GO/Alginate) composite.

The mechanism of these reactions may have occurred in a different scenario involving passive diffusion along a different channel, but the outcome would have been the same in both cases. Hydrogel scaffolds injected with the PGA outer shell-binding glutamate peptide domains are modeled mathematically. An extracellular matrix-derived scaffold is transported to the injured nerve neuron. The dynamics of the scaffold binding to peptides, free scaffold diffusion depending on glutamate receptor uptake, and enzymatic matrix degradation—driven by the targeted catalytic cleavage of the poly-glutamic acid outer shell—are all captured in this model. This study also looked at how changing the binding kinetics and the rate of enzymatic matrix degradation affected the release of scaffolds, in case there were unpredictable problems with the glutamate receptors. This system has the capacity to transport protein scaffolds and regulate their biological function.

These studies examined the possibility that scaffold-binding peptides, which are covalently immobilized to the nerve cell, may restore injured neural tissue and provide neuronal resistance to glutamate-induced damage. One of the peptide fragments’ amino acid sequences was used to manufacture the damaging compound, and the scaffold was bound to the immobilized peptides. Release from such a system can occur via two distinct mechanisms: the dissociation of a scaffold from matrix-bound peptides and the diffusion of a free scaffold from the matrix, or the proteolytic degradation of the nerve cell–matrix by the enzyme. The transglutaminase activity of Factor XIIIa forms a covalent bond between the scaffold-binding peptide and the fibrin matrix. Scaffolds are attached to the peptide through electrostatic interactions. Both free and bound peptides are subject to binding (and dissociation) between the scaffold and the matrix. The degradation of the fibrin matrix by plasmin frees up bound species [62]. The first release mechanism described above is passive and occurs in the presence or absence of cells, whereas the second and third release mechanisms are active and occur only in the presence of cells.

A mathematical model was created to characterize the uptake and diffusion of the scaffold from peptide short-chain matrices incorporating covalently immobilized scaffold-binding peptides with different affinities. The model was used to calculate the impact of scaffold-binding affinity and nerve–cellular matrix degradation on the rate of scaffold binding to glutamate receptors on domain surfaces. The model was intended to forecast the range of affinities and scaffold-to-binding site ratios that would be permitted to alter the rate of scaffold absorption by modifying the binding site’s affinity. From this, the affinity-based scaffold delivery system’s previously constructed model was enhanced [63].

**Scheme 1:**
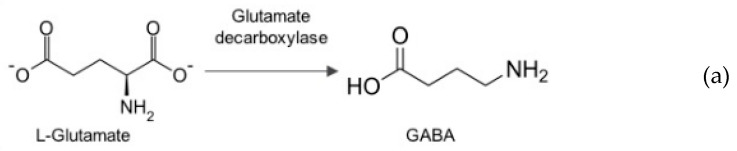
Glutamate decarboxylase reaction.

The model considered five species: an immobilized peptide attached to the matrix (mp), a free (unbound) peptide (p), an immobilized scaffold–peptide complex bound to the matrix (mpSC), and a free (unbound) scaffold–peptide complex (pSC). These species are part of a reaction network in which matrix breakdown releases degradable substances, resulting from the binding and dissociation of the scaffold and peptides. An expression that denotes matrix degradation brought on by enzymes that are triggered by nerve cells was added to a mathematical model. The enzyme that was responsible for matrix degradation is the enzyme that had already demonstrated the model reactions; that is, glutamate decarboxylase, which converts glutamate to GABA, which the scaffold releases into the nerve cell. Using glutamate decarboxylase catalyzes the irreversible β-decarboxylation of l-glutamic acid to γ-aminobutyric acid (GABA) and carbon dioxide (Reaction a, Scheme 1):

After a spinal cord injury, it is recommended to increase GABA levels since this neurotransmitter has been shown to suppress inflammation [64]. A malfunctioning GABA receptor is a strong possibility in this case. Research has demonstrated that the addition of chitosan to the produced hydrogel significantly enhances both GABA synthesis activity and concentration. This is evidenced by experiments involving the germination of brown rice in a chitosan/glutamic acid solution, showing increased GABA levels as a result [65].

**Scheme 2:**
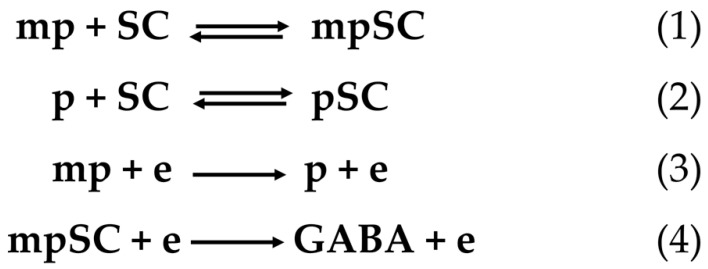
Reaction steps of scaffold degradation and dissociation processes.

The model consists of two components. The first part employs the batch reactor type within the reaction engineering interface to simulate a system where the environment is uniformly mixed, thereby eliminating any assumptions of spatial dependance. The objective of this section is to analyze the response rate of the system. In contrast, the second part introduces spatial dependence through the reaction engineering link. It uses the transport of diluted species into the porous catalyst interface to investigate how scaffolds migrate from the biomaterial to the area impacted by nerve endings. This part simulates four reactions that encompass scaffold degradation and dissociation processes, which are represented in this reaction scheme (Scheme 2):

To simulate nerve regeneration for the purposes of this investigation, a cylindrical structure measuring 12 mm in length and 3 mm in radius was modeled in the nerve cell–matrix. It was hypothesized that diffusion occurred in both the axial direction (y) and the radial direction (x). It was assumed that the network’s responses were all in balance before the onset of release. Additionally, it was assumed that the release from the cylinder would be axially and radially symmetrical and that there would be no delivery system components outside of the fibrin cylinder to begin with.

Initial Conditions: The following equations describe the model’s initial condition, in which all reactions are in equilibrium, and no matrix degradation has occurred [62]:

Concentration (cmp and cmpSC = 0).
Concentration of Enzyme = 2 × 10^−6^ [mol/m^3^]
Concentration of peptide = 0 [mmol/m^3^]
Concentration of SC = 0 [mol/m^3^]

### 2.5. Diffusion Coefficients

With regard to the diffusion coefficient, D_i_^k^ (m^2^/s), for species i in medium k, D_i_^k^ (mol/(m^3^·s)) is the rate expression for volumetric reactions (Equation (1)) of species i in domain k that solely involve bulk species. Matrix-bound species are mpSC and mp (from the reaction above) in the biomaterial. D^φ^_i_ is the surface reaction rate (mol/(m^2^·s)), and S_sa_ is the specific surface area of the biomaterial (1/m):

(∂c_i_)/∂t + ∇·(−D_i_^k^ ∇c_i_) = R_i_^k^ + R_s,i_ S_sa_ (1)

The species water diffusion coefficient was calculated using Equation (2). In this expression, M^w^ is the molecular weight of the species, and A is a constant having the value of 260 cm^2^/(s·Dalton):

D_i_^WaN^ = A·M^W−1/3^ × 10^7^ (2)

Based on the connection established by Saltzman et al. for protein diffusion in porous hydrogels [66], the diffusion coefficients of the different species in the gel were determined by multiplying D_i_ water by (0.9). Species diffusion coefficients are shown in Table 1. Using the calculated molecular weight (Table 2) for these species—which reflect bulk average estimates that are standard in contemporary tissue engineering literature—provides a clearer rationale for our upscaling strategy. The units have been converted from cm^2^/s to m^2^/s so that the modeling program can function correctly with the geometry we specified. Enzyme-catalyzed matrix degradation is described by Michaelis–Menten kinetics (Equation (3)), denoted by the rate constants RMMmp and RMMmpSC.

Enzyme-catalyzed matrix degradation is described by Michaelis–Menten kinetics. The rate expressions for the reactions are given by Equation (3):

Rsc2 = −kf1·csc(cmp·ssa + cp) + kr1(cmpsc·ssa + cpsc); Rp2 = −kf1·csc·cp + kr1·cpsc + RMMmp; Rpsc2 = kf1·csc·cp − kr1·cpsc + RMMmpsc; Rmp2 = −kf1·csc·cmp + kr1·cmpsc − RMMmp; Rmpsc2 = kf1·csc·cmp − kr1·cmpsc − RMMmpsc(3)

The breakdown of the nerve matrix by the enzyme was characterized using Michaelis–Menten kinetics. Table 3 mentions the reaction rate constants for biomedical species. Disparate breakdown will be indicated by a phrase that refers to the enzyme activity (Units/mL) in a certain volume [69]. KM and Vmax are constants for a certain enzyme [59]. The equation of Michaelis–Menten first order describes the concentration of the SC substrate that was found in the smallest amount c << KM (Equation (4)):

V_ma_^x^ = K_m_ × [degradable species (SC)] (4)

The loss of mp sites and the appearance of p species are characterized by RMMmp. The loss of mpSC locations and the emergence of pSC species are chronicled by RMMmpsc. The maximum rate is denoted by Vmax, and the Michaelis–Menten constant is denoted by KM. The rate expressions are based on the fact that only dissociation/association processes take place in the cellular zone (k = 1) and the surrounding media (k = 3).

Boundary Conditions: In this study, the axial symmetric boundary conditions were used, while the other boundaries were symmetric. The coefficients of diffusion and the rate constants are taken from published studies.

**Figure 6:**
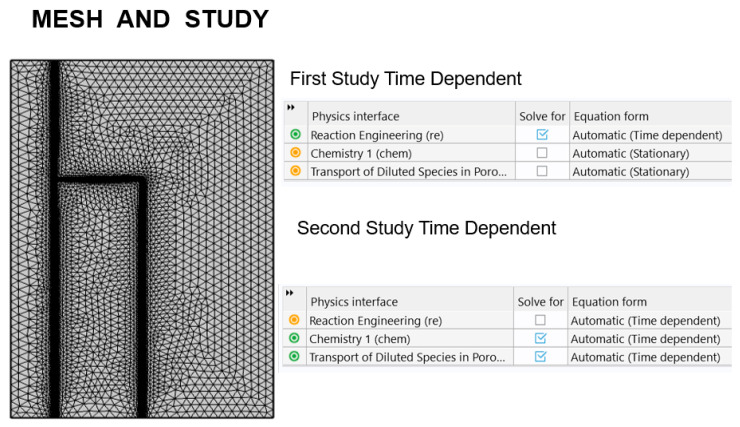
Release scaffold modeling spinal cord tissue regeneration mesh and study.

Mesh and Study: The model’s mesh (Figure 5) exhibits both an inner and an outside boundary condition. A finer mesh, especially towards the inside border, allows for a better he examination of the transporter’s chemical reaction. This section required two components with three time-dependent factors: physical interface studies (Figure 6), reaction engineering to define all chemical reactions that occurred using the study parameters from literature, and the reactions’ constants and styles, whether reversible or irreversible. A study of chemistry to investigate the molecular weight and function as species properties was conducted to investigate how different species move about within a nerve cell through passive diffusion, depending on whether or not the medium is porous.

## 3. Results

### The Release Scaffold Modeling Spinal Cord Tissue Regeneration

**Figure 7:**
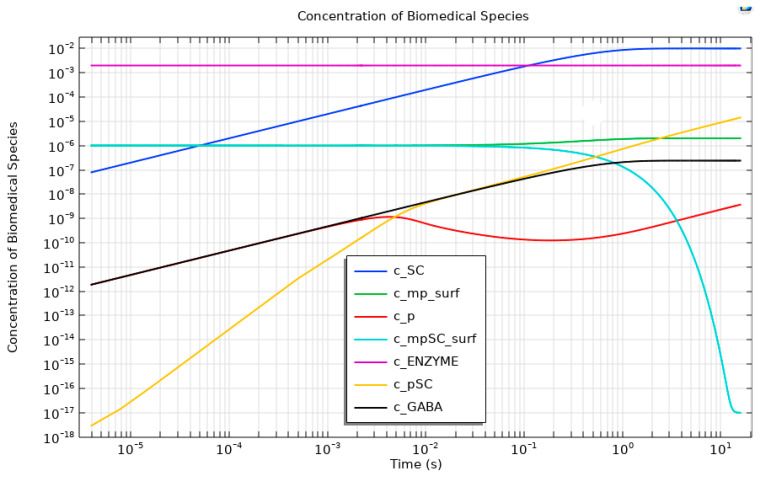
All species concentrations—SC, p, SCp, mpSC, mp, GABA, and Enzyme (in mol/m^3^)—as a function of time (s).

The data presented in Figure 7 clearly illustrate the impact of enzyme degradation. Over time, an increase in free peptide species (p and pSC) was observed, while the levels of matrix-bound peptide species (mp and mpSC) diminished. After around 16 s, the matrix (mpSC) had completely degraded. The pharmaceutical and peptide species exhibited the same association/dissociation kinetics. Additionally, the steady-state concentration of the scaffold remained unchanged regardless of whether the peptide was attached to a matrix or not. GABA concentration rose with time, reaching a plateau when scaffold concentration leveled out. It was observed that the levels of the enzymes remained constant.

**Figure 8:**
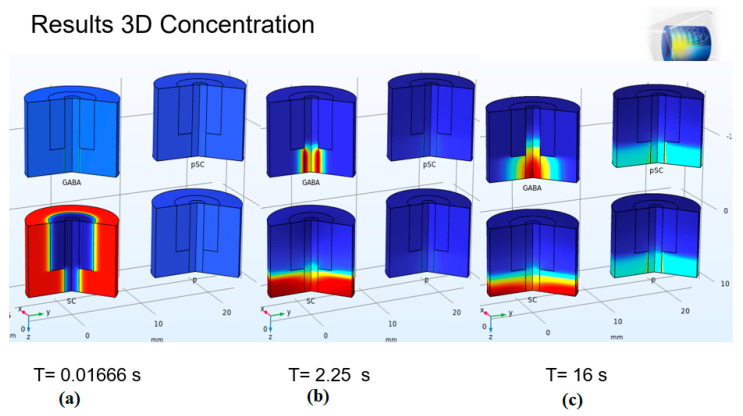
Representation of 3D bulk concentration (*p*, *SC*, pSC, and GABA) at different times: (**a**) 0.01666 s, (**b**) 2.25 s, and (**c**) 16 s.

**Figure 9:**
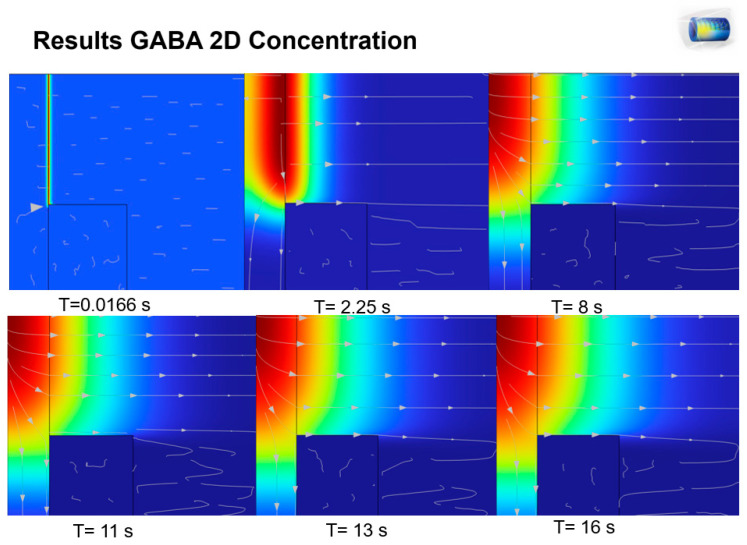
Representation of GABA concentration at different times: 0.0166 s, 2.25 s, 8 s, 11 s, 13 s, and 16 s.

By solving the space-dependent mass balances in (Equation (3)), the distributions of species concentrations throughout time are demonstrated. Figure 8 shows the total bulk species concentration, and Figure 9 represents bulk concentrations (peptide, scaffold, pSC, and GABA) at different times. The enzyme has its origin in the nervous system. The results display the overall drug release, making it apparent that matrix degradation targets the injured cellular area for drug delivery.

The presence of GABA in the nerve cells, as shown in (Figure 9), provides conclusive proof that the scaffold was taken up by astrocytes and destroyed by the enzyme at the nerve surface. There are several positive mechanisms involved, such as the astrocyte’s uptake of glutamate, which helps prevent further damage to nerve cells. Additionally, nerve cells can relocate to their proper location after injury.

**Figure 10:**
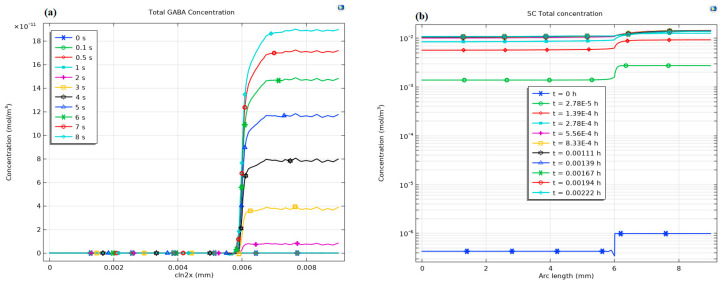
Concentration maps showing the distribution of total concentration across the module domain: (**a**) GABA total concentration, (**b**) scaffold (SC) total concentration.

Distribution maps of total concentrations of the scaffold and GABA are shown in Figure 10. The results of a module-wide concentration study revealed that GABA first appears near the module’s surface, where concentrations of the dissociated scaffold are highest.

**Figure 11:**
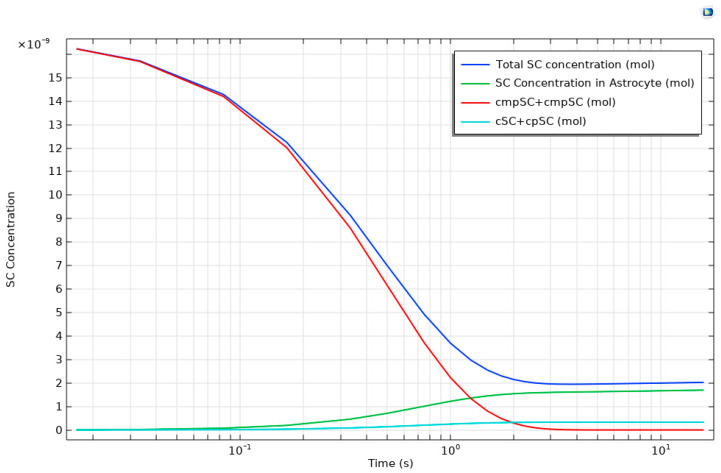
Total scaffold concentration.

In Figure 11, total scaffold concentration indicated a reduction in the scaffold–peptide binding matrix (mSCp and mSCp) within the ECM due to the impact of the degrading matrix. At the same time, there was a significant increase in SC in astrocytes and a slight increase in SC + pSC in nerve cells. As illustrated in Figure 11, the total scaffold concentration exhibits a sharp decline within the extracellular matrix due to ongoing enzymatic degradation. Concurrently, the accumulation of SC within the astrocyte domain confirms the successful targeted migration and internalization of the scaffold over the observed time scale.

## 4. Discussion

**Composite Properties and Neural Tissue Engineering:** The unique properties of composites have been highlighted in past studies, particularly their ability to assist cells in penetrating the scaffolds due to rGO’s porous nature. As such, the novel approach to nerve tissue treatment, using graphene-based materials, has gained traction. These materials exhibit exceptional physical properties, good conductivity, and the capability for connectivity with neurons and neuronal circuits. Next-generation materials for regenerative neurocore, which include graphene-based materials, hold significant promise for restoring neural tissue. Researchers are optimistic that the innovative strategies being developed, incorporating topographical and biomedical signaling, will change the lives of millions of paralyzed patients and clarify the tremendous neural tissue engineering (NTE) microfiber-scaffold potential for repairing nerves. In this context, green methods for producing rGO composite scaffolds have demonstrated remarkable biocompatibility and bioactivity. However, one shortcoming is their reduced plasticity, which impairs their NTE applications. Hydrogel scaffolds are being used to enhance the biocompatibility of graphene microfibers by covering them with a natural biocompatible polymer–poly-glutamic acid layer.

**Printability of Chitosan and PGA Hydrogel:** To test the printability of the chitosan and PGA hydrogel, researchers used both numerical simulations of extrusion-based chitosan 3D printers and experimental methods. The inner fluid, or dispersive phase (chitosan ink), entered the nozzle through a vertical channel, and the continuous phase, or air, impinged on the dispersive phase via side channels, bringing the two phases into diametrical contact. The 3D-printed products made from chitosan and PGA varied depending on the settings used. Based on the two-phase flow rate ratios of the entering ink fluids from the nozzle, the extrusion formation may speed up. When the velocity and the viscosity rate are increased, the filament becomes longer. Results from the 3D printer indicate that when the volume fraction increases, the extrude breakup time from the nozzle (period of extruding creation) decreases. The simulation and experimental assessment findings may be used to speed up the printing process while decreasing the cost of preparing PGA and chitosan hydrogel. Therefore, the results of this study may be used in the following processes of drug delivery for tissue engineering and the creation of chitosan and PGA hydrogels.

**Hydrogel Delivery System:** In the last step before the medication reaches the body, hydrogel delivery using microfluidic devices combined with in situ injection of a modeled water-soluble hydrogel offers the potential for controlled medication dosing and safe storage of the produced medication. In this study, the hydrogel scaffold operates as a core–shell or reservoir system, with the medication being the rGO nanocomposite scaffold and the outer shell material (PGA). This module simulation might be utilized to accurately simulate the injection of the manufactured drug at the desired concentration of the outer shell PGA. Drug flux from predominantly diffusion-controlled delivery systems had been quantitatively represented using relatively simple mathematical calculations. Fick’s law of diffusion is fundamental in understanding drug diffusion, especially in scenarios where no driving force is present. The concentration of the hydrogel is entirely controlled by the water droplet’s forward velocity and the mole fraction of the hydrogel in motion. By leveraging COMSOL simulations, it is possible to accurately determine the optimal velocity and duration for administering a specific dose of medication, ensuring effective delivery.

**Scaffold Delivery Systems:** Scaffold delivery systems designed to reinstate critical signals that are present during development but absent in adulthood—signals believed to aid in tissue regeneration—were integrated into materials through a combination of molecular biology and engineering techniques. Glutamate binding sites (in the form of covalently attached peptides) were included in these materials to produce an ECM-mimetic material that can preserve the hydrogel and/or rGO composite to prevent their degradation. There are no currently available rational strategies for selecting domains with varied glutamate–peptide affinities. To identify short peptide sequences with varying affinities for poly γ-glutamic acid (PGA), the outer surface of the prepared hydrogel was used. The logical design of ECM-mimetic materials for scaffold distribution was subsequently made possible by the mathematical modeling of the scaffold’s binding kinetics and diffusion characteristics. Regulating the release rate is achieved by “completely” separating the scaffold where the depot is situated at the core of the dosage form, and the polymer acts as a membrane surrounding this depot. The enzyme reaction is modeled using Michaelis–Menten kinetics. An injectable rGO-based nanocomposite scaffold hydrogel model, with an outer shell containing L-glutamic acid, demonstrated that the primary effect is the enhanced absorption of glutamate from the extracellular space by astrocytes. This is comparable to the observations recorded in lampreys following just one day of spinal cord disruption. The hydrogels eventually degraded in vitro, releasing an rGO-based nanocomposite scaffold in situ that promoted better axon ingrowth, lasted longer, and had the potential to conform to the shape of the defect, thereby restoring tissue continuity and lessening the dramatic contraction/deformation of the scaffolds. The study presented GABA as evidence indicating that the matrix effectively delivered the scaffold to the injured nerve cell. It concluded a novel concept: by forcing all glutamate species to exit the extracellular matrix (ECM), they are rapidly taken up by the astrocyte to halt their neurotoxic effects. This process occurs when the PGA, the outer shell of the rGO composite, is degraded enzymatically, resulting in the production of GABA and CO_2_. To restore the nerve cell’s full functionality, it is recommended to implant MSCs within the degradable composite to start its “growth journey” toward compensating for the loss of nerve cells. This means that tests on living organisms (in vitro and in vivo) may begin.

The release kinetics within the scaffold delivery system were modeled assuming standard physiological conditions (a temperature of 37 °C and a neutral pH of 7.4) to accurately reflect the in vivo environment of the spinal cord.

Because formal mesh convergence and sensitivity analyses were not conducted in this initial study, the numerical stability of the long-term concentration profiles introduces a degree of uncertainty. Comprehensive parameter sensitivity testing and direct validation against experimental in vitro data remain the next necessary steps.

**Model Assumptions and Limitations:** While this framework provides insights into hydrogel-mediated transport, certain macroscopic approximations present a challenge that requires further consideration. In this exploratory model, glutamate and glutamine were treated with analogous transport kinetics to maintain computational tractability. Future high-resolution models should account for the distinct electrostatic interactions of the negatively charged glutamate within the extracellular matrix. Additionally, the application of synaptic-scale kinetic rate constants to the bulk biomaterial assumes a well-mixed isotropic continuum, representing a necessary effective medium approximation for tissue-scale porous flow. It is important to acknowledge certain simplifications in the current model. Glutamate and glutamine were treated with similar diffusion characteristics; however, future iterations should account for their distinct molecular weights, charges, and receptor interactions. Additionally, the assumption of a constant NH_3_ concentration simplifies the complex glutamate–glutamine cycle and potential ammonia toxicity. The astrocytic uptake mechanism was also generalized, not distinguishing between specific astrocyte subtypes or varying injury states. These simplifications were necessary for computational feasibility but represent key areas for future refinement.

**Pathophysiological Links**: The simulated concentration profiles provide critical insights into the pathophysiological processes following SCIs. The rapid reduction in extracellular glutamate concentration, as shown in the model, directly correlates with falling below the excitotoxicity threshold (0.05–0.10 mM), thereby potentially mitigating secondary neuronal death. Furthermore, the sustained release of GABA may contribute to modulating the inflammatory response, which is a precursor to glial scar formation. While the model draws qualitative inspiration from the robust glutamatergic system recovery observed in lampreys, future quantitative studies are required to directly compare lamprey and mammalian kinetic parameters.

## 5. Conclusions

This study discovered an innovative way to deliver rGO nanocomposite safely to the injured spinal cord within 72 h without triggering the immune system. The rGO-based nanocomposite scaffold was simulated using COMSOL. The scaffold was developed through a simple electrostatic layer-by-layer self-assembly nanosphere approach, combining rGO as a core, chitosan as an inner layer, and poly-glutamic acid as an outer layer into a hybrid injectable hydrogel model. The hydrogel safely travels inside the spinal cord injury and prevents further neural cell death after spinal cord injury, by increasing astrocytes’ glutamate uptake from the extracellular matrix, which is similar to what is seen in lampreys after the spinal cords are disrupted by injury.

This work provides context for the specific COMSOL Multiphysics features the reader would want to evaluate in light of its own modeling goals. These features include the simulation of in vivo drug release, as well as a wide range of diffusion applications across multiple domains. The application of COMSOL software to this case yielded outstanding results in passive diffusion. It looked at the differences in density between neurons with different functions to show how therapeutic substances move through living things and the different speeds at which these substances pass through nerve cells and other porous cells. We need to apply more advanced simulation software to enhance the success of this study, as many simulation cases were not applicable. Moreover, these simulation results highlight the critical need for complementary experimental work to validate the models and ensure their relevance and accuracy in real biological systems.

## 6. Recommendations

The synthesis of rGO–alginate composite has not been considered in the model initially; composite fibers could be produced by electrospinning. Due to their inherent similarity to the microstructure of real nerve tissues, fiber-based scaffolds have become widely used in tissue engineering. Electrospinning is widely used as a method for making continuous fibers. Electrospinning’s benefits include (i) simple processing, (ii) the opportunity for massive production, and (iii) the accessibility of cutting-edge modes [71]. Jin et al. [72] used this concept to generate a polymer core-CNS in order to create an rGO–alginate composite core–shell nanofiber, exhibiting an extensive network of π-conjugated bonds formed on the rGO shell layer’s surface. For promoting endothelial cell proliferation, Wu et al. devised a layer-by-layer (LbL) technique to coat electrospun nanofibers that simulate vascular ECM. Modifying and controlling the porosity of the gel (ca. 99% ± 0.3%) by introducing GO and rGO into an alginate matrix results in holes of a consistent size from the gel’s surface to its inner core, which in turn promotes cellular activity. To achieve the ideal swelling index necessary for an effective scaffold, GO and rGO composite gels may also be used. The analysis of mechanical and electrical characteristics suggests that a 0.1 wt.% GO concentration was optimal.

The model does not account for binding processes or the rate of glutamate diffusion in spinal cord neurons. Although not yet demonstrated, it is possible that glutamate binds to the ECM or other solutes in a competitive manner. This limitation of the theoretical model could be addressed in future research. Since annulus fibrosus (AF) and nucleus pulposus (NP) cells differ in phenotypic expression [73], the KM and Vmax of glutamate in the theoretical model may vary between the two cell types. The solute kinetics model could be improved by incorporating changes in Michaelis–Menten parameters.

When designing a drug-delivery system, it is important to consider the biodegradability and biocompatibility of the materials used. It is essential to recognize the significant impact that degradation products and the rate of degradation—which were not addressed—have on the system. Biodegradable polymers break down into smaller, biocompatible compounds and then into carbon dioxide (CO_2_), nitrogen (N_2_), and water (H_2_O). This feature allows for the production of drug-loaded polymer particles that can contain the drug and release it gradually throughout the body [74].

## Figures and Tables

**Table 1:** Diffusion coefficients for all biomaterial types (SC, pSC, p, GABA, and e) in three domains (A: Astrocyte, E: Extracellular Matrix, and N: Nerve cell), calculated from [66] for protein diffusion in porous hydrogels.

Diffusion Coefficient	Value
Dp_A	1.1476 × 10^−7^ m^2^/s
Dp_E	1.2751 × 10^−7^ m^2^/s
Dp_N	1.1476 × 10^−7^ m^2^/s
De_E	4.91 × 10^−4^ m^2^/s
De_A	3.4667 × 10^−5^ m^2^/s
De_N	3.12 × 10^−5^ m^2^/s
DGABA_E	8.3 × 10^−13^ m^2^/s
DGABA_A	8.9371 × 10^−8^ m^2^/s
DGABA_N	8.0434 × 10^−8^ m^2^/s
DpSC_E	9.1 × 10^−6^ m^2^/s
DpSC_A	8.19 × 10^−6^ m^2^/s
DpSC_N	8.19 × 10^−6^ m^2^/s
DSC_E	8.2333 × 10^−6^ m^2^/s
DSC_A	7.41 × 10^−6^ m^2^/s
DSC_N	7.41 × 10^−6^ m^2^/s

**Table 2:** Molecular weight of all biomaterial species (SC, pSC, p, GABA, and e).

Biomaterial Species	Molecular Weight [kg/mol]	References
H_2_O	0.018	[62]
SC	9.5	[67]
Psc	10.5	[62]
GABA	0.10312	[68]
p	1	[62]

**Table 3:** Chemical reaction rate constants for biomedical species.

Rate Constant	Value	Expression	References
kf_mm	0.007336 1/s	Reaction rate constant, matrix-degradation following Michaelis–Menten kinetics	[62]
kf_d	5 × 10^6^ m^3^/(s·mol)	Forward reaction rate constant, Glutamate binding rate	[70]
Keq	0.00485	Glutamate–Glutamine (GLU-GLN) keq	[70]
Kb	10^−8^ m^3^/(s·mol)	Kinetic constant of GLU binding of Mglu	[70]
Ku	2 [1/s]	Kinetic constant of GLU unbinding of Mglu	[70]
kmGlu	0.3 [mol/m^3^]	GLU Michaelis–Menten constant	[70]

## Data Availability

The original contributions presented in this study are included in the article. Further inquiries can be directed to the corresponding author: aabouzieh@just.edu.jo.

## Abbreviations

The following abbreviations are used in this manuscript:

SCI	Spinal Cord Injury
rGO	Reduced Graphene Oxide
PGA	Poly γ-Glutamic Acid
ECM	Extracellular Matrix
GABA	Gamma-Aminobutyric Acid
GS	Glutamine Synthetase
CNS	Central Nervous System
NSC	Neural Stem Cell
GO	Graphene Oxide
MSC	Mesenchymal Stem Cell
BSCB	Blood–Spinal Cord Barrier
NTE	Neural Tissue Engineering
GLU	Glutamate
GLN	Glutamine
ATP	Adenosine Triphosphate
GAD	Glutamate Decarboxylase
NP	Nanoparticle
